# How can clinical trials expedite the process of answering treatment-related questions and reduce the number of participants needed?

**DOI:** 10.1192/j.eurpsy.2024.133

**Published:** 2024-08-27

**Authors:** R. Emsley

**Affiliations:** Institute of Psychiatry, Psychology and Neuroscience, London, United Kingdom

## Abstract

**Abstract:**

Patients and the research community need better and more cost-effective randomised trials. These are the ‘gold standard’ way of seeing if a new treatment works or not, and take years of effort involving lots of patients and funding. However, around half of trials fail to show that the new treatment is better than what it is being compared with. In cancer, this problem has been recognised. They use trial designs which test multiple treatments, and find out quicker answers to more questions. These ‘efficient trials’ are able to involve patients at a faster rate and to improve the chances of patients receiving a treatment that works. In mental health, the whole toolbox of trial designs is not being used. Sometimes there are valid reasons for this, but sometimes it is simply that researchers do not know about them – this talk will expand on the concept of ‘efficient trials’ in mental health, and present the opportunities and challenges to using these.

**Disclosure of Interest:**

None Declared

